# Meta-synthesis of rehabilitation experiences and needs of adolescent patients with mild traumatic brain injury

**DOI:** 10.3389/fneur.2025.1618811

**Published:** 2025-08-13

**Authors:** Cheng Cheng, Yue Yu, Chunjuan Yao, Hongyan Bian

**Affiliations:** ^1^Department of Neurology, Tianjin Huanhu Hospital, Tianjin, China; ^2^Department of Nursing, Tianjin Huanhu Hospital, Tianjin, China

**Keywords:** MTBI, adolescents, rehabilitation experience, qualitative research, meta-synthesis, evidence-based nursing

## Abstract

**Objective:**

This paper aims to systematically evaluate and integrate qualitative research on the experiences and needs of adolescent patients with mild traumatic brain injury (mTBI) during rehabilitation and to provide references for rehabilitation care strategies for such patients.

**Methods:**

PubMed, Web of Science, Embase, Cochrane Library, CINAHL, Scopus, Medline, PsycINFO, China National Knowledge Infrastructure, Wanfang Database, China Biomedical Literature Database, and VIP Database were searched for relevant qualitative studies until September 15, 2024.

**Results:**

A total of 863 documents were found, and after screening, eight documents were finally included, of which two were rated A and six were rated B in terms of quality. The 40 integrated results were summarized into 12 categories and combined into three integrated results. The primary themes included the symptoms exhibited by adolescents with mTBI and their impact on daily life; the needs and challenges of adolescents with mTBI during the rehabilitation period; and the self-management experiences of adolescents with mTBI during the rehabilitation period. Secondary themes included interference with daily activities; forced termination of hobbies and interests; academic difficulties; increased negative emotions; social withdrawal; obtaining academic accommodations; acquiring information and knowledge about the disease; help from family and friends; timely and effective treatment and rehabilitation; control of the environment; adjusting rest and activity strategies; and active self-healing.

**Conclusion:**

Medical staff should focus on the feelings, reported needs, and practical difficulties of mTBI adolescents during the rehabilitation period and formulate more personalized rehabilitation strategies for them.

## 1 Background

Mild traumatic brain injury (mTBI), also known as concussion, is the most common traumatic brain injury in clinical practice, covering approximately 70–90% of all brain injuries (1), with an incidence of 700/100,000 people per year (2). Hence, it is considered a crucial public health issue. Cranial brain injuries are generally classified into four types: mild, moderate, severe, and extremely severe. These are primarily distinguished based on five factors: time of loss of consciousness, neurological signs, neurological imaging, basic vital signs, and subjective symptoms. Specifically, mTBI is characterized by a coma duration of less than 30 min, a Glasgow Coma Scale (GCS) score of 13–15 after injury, no structural brain injury on neuroimaging, no positive neurological findings on neurological examination, stable vital signs such as pulse and respiration, as well as symptoms such as ecmnesia, headache, dizziness, tinnitus, nausea, and inattention (3). mTBI is also a common pediatric injury, and 19.5% of American adolescents have been diagnosed with mTBI (4). It influences multiple aspects, including physical activities, emotions, behaviors, and cognitive function. About 30% of mTBI adolescents experience symptoms for more than 4 weeks and even continue to suffer from it after the first year (5). Adolescence is a challenging and unique stage of life. Physically, adolescents enter a period of rapid growth accompanied by hormonal changes and the development of secondary sexual characteristics. Psychologically, adolescents experience significant mood swings, but their ability to regulate their emotions gradually improves as they begin to seek greater independence and autonomy. In terms of cognition, adolescents experience significant improvements in their thinking patterns and learning abilities. This period is also critical for adolescents to establish correct values, healthy behaviors, and lifestyles. Therefore, adolescent development is of great significance for future life. However, mTBI may affect adolescents’ developmental trajectories in multiple dimensions, yet this is often overlooked.

Multiple studies have suggested that proactive rehabilitation should be applied for adolescents who still manifest post-concussion symptoms (6–8). These rehabilitations are not limited to medical aspects but also extend to the educational and psychological aspects. Single qualitative studies are insufficient to comprehensively explain research findings. Therefore, this study aims to comprehensively reflect the experiences and needs of adolescents with mTBI during rehabilitation by summarizing relevant qualitative studies. This will provide a basis for developing more comprehensive and effective rehabilitation strategies.

## 2 Data and methods

### 2.1 Inclusion and exclusion criteria

According to the participants, interventions, comparators, outcomes, and study design (PICoS) recommended by the Joanna Briggs Institute (JBI) Evidence-based Health Care Center, literature inclusion and exclusion criteria were formulated.

Inclusion criteria:

1. Participant (P): mTBI individuals, aged 12–24 years old;
2. Interest of phenomena (I): life impacts, feelings, experiences, and needs of mTBI adolescents during the rehabilitation period;
3. Context (Co): rehabilitation environments for mTBI adolescents, including rehabilitation institutions, rehabilitation clinics, communities, families, and schools;
4. Study design (S): phenomenology, grounded theory, data analysis, and qualitative research.

Exclusion criteria:

1. Studies not published in the Chinese and English languages;
2. Literature with unavailable full texts or incomplete data;
3. Conference summary and reviews;
4. Published duplicates.

### 2.2 Retrieval strategy

PubMed, Web of Science, Embase, Cochrane Library, CINAHL, Scopus, Medline, PsycINFO, China National Knowledge Infrastructure (CNKI), Wanfang Database, Chinese Biomedical Literature Database (CBM), and VIP Database were searched for relevant qualitative studies until September 15, 2024. English search terms included “Brain Concussion/Brain Concussions/Cerebral Concussion/Cerebral Concussions/Commotio Cerebri/Intermediate Concussion/Intermediate Concussions/Mild Concussion/Mild Concussions/Mild Traumatic Brain Injury/Severe Concussion/Severe Concussions,” “Exercise Therapy/Rehabilitation Exercise/Exercise Rehabilitation/Rehabilitation Exercises/Therapy Exercise/Exercise Therapies/Therapies Exercise/Remedial Exercise/Exercise Remedial/Exercises Remedial/Remedial Exercises/rehabilitation,” and “Qualitative Research/Qualitative Studies/Interviews/NVivo/content analysis/descriptive study/exploratory/focus group/phenomenology/thematic analysis/grounded theory/qualitative research/qualitative study.” Chinese search terms were “mild traumatic brain injury/concussion/minor concussion/mild concussion/brain injury/mild brain injury/minor brain injury,” “exercise therapy/rehabilitation exercise/exercise therapy/rehabilitation/exercise/physical activity/physical activity/body movement/body activity,” and “qualitative research/experience/interview/feelings/ethnography/ethnography/exploratory research/focus group/phenomenology/thematic analysis/grounded theory/qualitative research.” By exemplifying PubMed, the search strategy was: #1(“Brain Concussion”[Mesh] OR Brain Concussions [Title/Abstract] OR Cerebral Concussion [Title/Abstract] OR Cerebral Concussions [Title/Abstract] OR Commotio Cerebri [Title/Abstract] OR Intermediate Concussion [Title/Abstract] OR Intermediate Concussions [Title/Abstract] OR Mild Concussion [Title/Abstract] OR Mild Concussions [Title/Abstract] OR Mild Traumatic Brain Injury [Title/Abstract] OR Severe Concussion [Title/Abstract] OR Severe Concussions [Title/Abstract]). #2(“Exercise Therapy”[Mesh] OR Rehabilitation Exercise [Title/Abstract]) OR Exercise, Rehabilitation [Title/Abstract] OR Exercises, Rehabilitation [Title/Abstract] OR Rehabilitation Exercises [Title/Abstract] OR Therapy, Exercise [Title/Abstract] OR Exercise Therapies [Title/Abstract] OR Therapies, Exercise [Title/Abstract] OR Remedial Exercise [Title/Abstract] OR Exercise, Remedial [Title/Abstract] OR Exercises, Remedial [Title/Abstract] OR Remedial Exercises [Title/Abstract] OR rehabilitation [Title/Abstract]). #3(Qualitative Research [Mesh] OR Qualitative Studies [Title/Abstract] OR Interviews [Title/Abstract] OR NVivo [Title/Abstract] OR content analysis [Title/Abstract] OR descriptive study [Title/Abstract] OR exploratory [Title/Abstract] OR focus group [Title/Abstract] OR phenomenology [Title/Abstract] OR thematic analysis [Title/Abstract] OR grounded theory [Title/Abstract] OR qualitative [Title/Abstract]). #4: #1 AND #2 AND #3.

Taking CNKI as an example, the Chinese search strategy was: #1: Post-concussion headache + sports-related concussion + exercise concussion + concussion injury + adolescent concussion + exercise-associated concussion + pediatric concussion + mild traumatic brain injury + brain injury; #2: Exercise therapy + rehabilitation exercise + kinesiotherapy + rehabilitation + rehabilitation training + rehabilitation treatment + rehabilitation nursing + rehabilitation guidance + early rehabilitation + sports activities + physical activity + body movement + physical exercise; #3: Qualitative research + qualitative study + experience + interview + perception + ethnography + exploratory research + focus group + phenomenology + thematic analysis + grounded theory + qualitative research. #4: #1 AND #2 AND #3.

### 2.3 Literature screening and data extraction

Literature screening, data extraction, and cross-checking were conducted by two researchers who were trained in qualitative research. Disagreements were resolved by a third researcher to determine eligible studies. Duplicate literature was removed by EndNote X9 software. Titles and abstracts were reviewed for initial screening. Studies with mismatched research methods, subjects, or topics were excluded. Full texts were read to determine the included literature. Extracted data consisted of authors, publication date, country, research methods, subjects, phenomena of interest, contextual factors, and main results.

### 2.4 Quality assessment

Two researchers independently appraised the quality of the included literature based on the JBI Evidence-Based Health Care Center’s qualitative quality assessment criteria (2024 version) (9). The evaluation scale consisted of 10 items, each rated as “yes,” “no,” or “unclear.” Studies fully meeting the criteria were classified as Grade A, partially meeting as Grade B, and not meeting at all as Grade C. Discrepancies in evaluation results were resolved by a third researcher. Ultimately, studies rated as Grade A and B were selected, while no publications were excluded due to Grade C criteria.

### 2.5 Analysis method

A pooled meta-synthesis approach was applied. After receiving training and mastering the philosophical thoughts and methodologies of qualitative research from the publication Research Methods in Chinese Nursing (10), researchers repeatedly reviewed the literature to summarize the research findings, form new categories, and analyze their relationships. The results were then further synthesized and integrated.

## 3 Results

### 3.1 Literature retrieval and screening results

**Figure 1:**
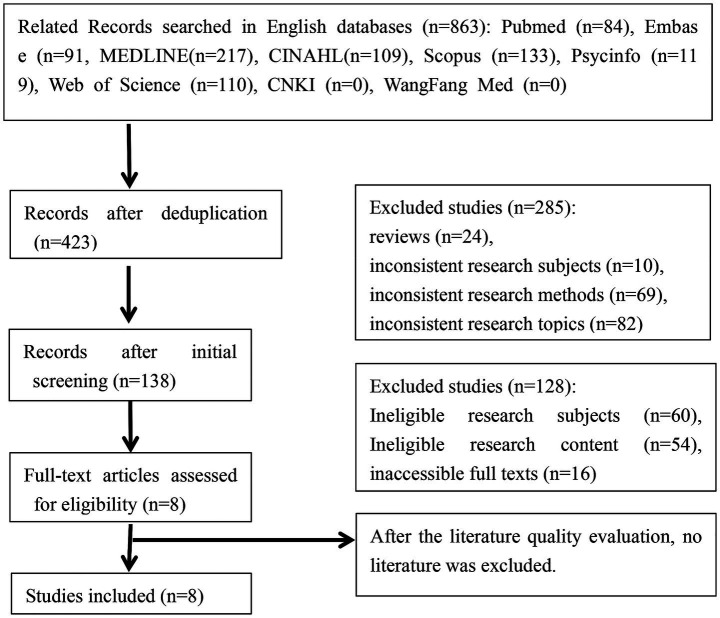
Literature screening process.

A preliminary search identified 863 relevant articles, with 8 ultimately included (11–18). (Figure 1).

### 3.2 Basic characteristics of included literature and quality assessment of methodology

8 English articles were included, with research settings of hospital outpatient departments, independent clinics, academic institutions, online platforms, and communities. Among the included articles, 2 studies were rated as Grade A in quality assessment, while the others were rated as Grade B. Table 1 lists the basic characteristics of the included literature, and Table 2 lists the results of the methodological quality assessment.

**Table 1:** Basic features of the included literature (*n* = 8).

Studies	Publication date (Year)	Country	Method	Subject	Interest of phenomena	Context	Main outcome
Shepherd et al. (11)	2024	Canada	Phenomenological study	20 adolescents (14–18 years old) with post-concussion syndrome	Experience of returning to school	School	5 topics: concussion symptoms affect adolescents’ academic performance; obtaining academic accommodations helps adolescents return to school; having supportive and understanding friends, family, and teachers aids adolescents in returning to school; communication among school stakeholders is necessary but often missed; anxiety, frustration, and sadness occur during the return-to-school process.
Moen et al. (12)	2022	United States	Phenomenological study	8 children and adolescents (11–18 years old) with concussions	Returning to school after a concussion and its impact on future career participation	Community	3 topics: different concussion experiences; knowledge is key to concussion management; concussion impacts professional engagement.
Quatman-Yates et al. (13)	2022	Canada	Phenomenological study	27 adolescents (13–17 years old) with MTBI	Effect of cognition on family functioning and rehabilitation activity levels during the first 4 weeks of post-MTBI recovery	Hospital outpatient department	3 topics: disruption of routine activities; considerations for damage management; positive and negative influencing factors
Graves et al. (14)	2020	United States	Phenomenological study	18 adolescents (10–18 years old) with concussions	Financial difficulties faced by families after a concussion	Unclear	In addition to direct medical insurance costs, families of adolescents with concussions face numerous indirect expenses, such as transportation, tutoring, and financial losses due to parental absenteeism. The cost of concussion treatment varies significantly among different participants.
Kita et al. (15)	2020	Canada	Data analysis Stepwise deductive induction	10 girls (14–19 years old) with a history of concussion	High school girls’ lived experiences of receiving social support during concussion recovery	Unclear	4 topics: emotional challenges with peers; practical challenges of returning to school; valuable and meaningful support; role of knowledge in facilitating meaningful support
Hodges et al. (16)	2019	Canada	Phenomenological study	10 adolescents (14–17 years old) with concussion	Experiences and self-management strategies of adolescents after concussion	Clinic	Adolescents reported that concussions disrupted their daily lives, particularly academic and learning activities. The most common self-management strategies were rest, environmental control, and the use of motivational thoughts and activities.
Hunt et al. (17)	2017	United States	Phenomenological study	8 high school football players with sports-related concussions but fully recovered	Psychological impact of cognition and rest on adolescents with sports-related concussions	Unclear	2 topics: team sports as social support; boredom from lack of social activities is detrimental to recovery.
Gagnon et al. (18)	2008	Canada	Phenomenological study	15 adolescents with MTBI	Service needs for adolescents after mTBI	Unclear	Adolescents hope to receive prompt treatment from experts really caring for them and recognizing their different needs from younger children. Strengthening communication between the healthcare and school systems beforehand can help them return to demanding academic activities.

**Table 2:** Methodological quality assessment of the included literature (*n* = 8).

Literature included	(1)	(2)	(3)	(4)	(5)	(6)	(7)	(8)	(9)	(10)	Quality assessment (Level)
Shepherd et al. (11)	Y	Y	Y	Y	Y	Y	Y	Y	Y	Y	A
Moen et al. (12)	Y	Y	Y	Y	Y	Y	Y	Y	Y	Y	A
Quatman-Yates et al. (13)	Y	Y	Y	Y	Y	Y	Y	Y	Un	Y	B
Graves et al. (14)	Y	Y	Y	Y	Y	N	N	Y	Y	Y	B
Kita et al. (15)	Y	Y	Y	Y	Y	N	N	Y	Y	Y	B
Hodges et al. (16)	Y	Y	Y	Y	Y	N	N	Y	Y	Y	B
Hunt et al. (17)	Y	Y	Y	Y	Y	N	N	Y	Y	Y	B
Gagnon et al. (18)	Y	Y	Y	Y	Y	Y	Y	Y	Un	Y	B

Note: (1) Whether the philosophical foundation and methodology are consistent; (2) Whether the methodology aligns with the research questions or objectives; (3) Whether the methodology is consistent with the data collection methods; (4) Whether the methodology aligns with the representativeness of the data and data analysis; (5) Whether the methodology is consistent with the interpretation of results; (6) Whether the researcher’s status is explained from the perspective of cultural background and values; (7) Whether the influence of the researcher on the study or the influence of the study on the researcher is explained; (8) Whether the study is representative; whether it adequately represents the subjects and their views; (9) Whether the study complies with current ethical standards; (10) Whether the conclusions are derived from the analysis and interpretation of the data. (Y stands for yes, N for no, and Un for unclear).

### 3.3 Results of meta-synthesis

The 50 integrated results were summarized into 12 categories and combined into three integrated results.

#### 3.3.1 Integrated outcome 1: symptoms and impact on the life of adolescents with mTBI

##### 3.3.1.1 Category 1: Interfere with daily life

Daily activities mainly refer to the necessary activities that people perform every day to meet their daily needs, including basic daily living and functional daily living. In basic daily life, sleep problems were often mentioned, with adolescents with mTBI frequently describing themselves as tired, insomniac, or hypersomniac (“I feel sluggish and want to sleep” (16)). In functional daily life, the decline in memory and reading ability is most prominent (“There is a gap between reading it and my brain understanding it”) (16), which can lead to difficulties in daily organization, storage, and finding items.

Some adolescents noted that similar locations to injury-related scenarios caused them distress and avoidance, making daily commuting challenging, Additionally, other family members needed to take care of them, leading to increased household financial costs, like transportation expenses, medical bills, and reduced family productivity (“Because my parents have their own business, you know, going to the clinic is a whole day’s torture”) (14). The impact of daily life is a test for adolescents themselves, as well as for other family members. It is essential for the community in which the family lives to provide medical services and assistance.

##### 3.3.1.2 Category 2: Forced reductions in recreational and sports activities

Adolescents have a wide range of interests and hobbies, which are not only for leisure and entertainment but also a means of self-expression and self-actualization. They are also an important way to enhance a sense of belonging and identity within a group. After mTBI, adolescents reduced their activities in hobbies due to physical symptoms such as headaches, dizziness, and sensitivity to sound and light. They were often advised to stop or decrease the time they spend watching TV, playing video games, listening to music, and playing instruments. (“I started dancing at four years old, and I was the lead in the company’s dance performances, but now I cannot participate anymore”) (16). Additionally, adolescents had to change their sports habits. Fearing further injury, they gave up the sport they loved, which even affected their future career choices (“I have earned my black belt in martial arts—it is my peak, but I have to stop there. I love the sparring part but I really cannot do that anymore. I quit martial arts, and it changed my life”) (12). These hobbies are often discontinued based on the advice of healthcare professionals. However, there is no mention of when or how they should be resumed. This requires a multidisciplinary team of professionals, such as sports coaches, music teachers, and healthcare professionals, to develop a strategy together. However, such collaboration is clearly not common.

##### 3.3.1.3 Category 3: Academic struggles

During adolescence, as academic workload and competitive pressure increase, academic performance gradually becomes a focal point of concern for individuals, families, schools, and society. Therefore, adolescents with mTBI often rush to return to school under academic pressure, but the results are often unsatisfactory. First, the school environment can exacerbate physical symptoms. (“The school lights are especially bright and are hurting my eyes. I have to go home an hour after arriving at school”) (12). Even when they persist in attending school, physical discomfort and lack of concentration also make classroom learning inefficient (“Taking notes, looking at the blackboard, shifting focus back and forth, is a bit difficult”) (11). When unable to persist in class, adolescents frequently visit the nurse’s office for breaks (“If I cannot focus, I just wander around for the entire class. Sometimes I go there just to get some medicine”) (14). Meanwhile, mTBI leads to a decline in learning abilities, including memory loss, slowed reactions, and reduced reading and thinking skills (“I cannot remember things, and my brain mixes up words”) (11), resulting in brain fog when handling school-related tasks. Furthermore, widespread digital learning tools worsen physical discomfort, further resulting in poor learning efficiency (“Looking at a smart tablet, or trying to read a sentence in a book or script, also makes my eyes very tired and worsens my headaches.” (15)). Finally, when faced with piled-up assignments and tasks, adolescents fell more pressure and are reluctant to rest (“Since it is the end of the school year, I do not have much time to catch up on about three weeks of missed work…; I have to finish everything before finals”) (13), which in turn worsens mTBI symptoms, creating a vicious cycle. Clearly, the school administration has not provided sufficient support for adolescents returning to campus and has failed to establish a unified and feasible policy.

##### 3.3.1.4 Category 4: Increased negative emotions

Negative emotions are emotions with negative valence, mainly including anxiety, fear, and anger. Compared with their physical development, adolescents’ psychological development is relatively slow, which means that they are still in a semi-mature state. When encountering changes and failures, they are more likely to feel frustrated and experience negative emotions. Although some believe that they are not affected by mTBI (“I just put one more thing to my experience lists, but I do not suppose it changes who I am”) (12), most adolescents are caught up in negative emotions, such as anxiety, fear, sorrow, and self-doubt. The pressure of failing to complete one’s academic work is the primary source of anxiety and fear (“I’m falling behind, I have to retake exams, so I have so much to do, and it is very overwhelming for me”) (15). At the same time, to obtain more information or convenience, adolescents need to communicate with schools, medical institutions, and other organizations during their recovery period, but the process is often fraught with obstacles, which further exacerbate their psychological burden (“If I cannot get an exemption from school in time, I’m worried I’ll have to write it, and I will not do well”) (11). Moreover, concerns about future life and the fear of reinjury often trouble adolescents (“‘My goodness, will I get another concussion?’ It makes me very worried about hitting my head again and how it might affect my memory and concentration”) (12). These unresolved pressures trap adolescents in various negative emotions, leaving them feeling overwhelmed. (“I’ll become very, very sad for no reason”) (16). Emotional issues are common, and internalizing them can easily lead to mental health problems. However, professional psychological assessments and guidance are rarely mentioned, and when faced with negative emotions, adolescents often find themselves “fighting alone.”

##### 3.3.1.5 Category 5: Social withdrawal

Social withdrawal refers to passive, evasive, and isolated behaviors in social situations. After mTBI, adolescents began to actively or passively reduce or even avoid social activities due to physical and psychological changes (“I want to stay away from everyone, without worrying about anything or anyone”) (16). Some adolescents describe feelings of exhaustion when participating in social interactions (“Talking to people makes me so tired that my entire social life is ruined. I just cannot talk to anyone”) (13). They are misunderstood but unable to defend themselves (“Oh, you are just doing this to avoid school…” It is too difficult… So I have not said much yet”) (13). Many adolescents feel awkward and embarrassed about bothering others (“It is like a compulsion; they do not fully understand the situation… It is more like they are robots. It is like they read online ‘This is what I should do,’ and then they do it”) (9). Socializing is an essential part of life. Can we provide a social platform where adolescents with mTBI feel comfortable? For example, they can participate in screen-based recreational social activities via their mobile phones to avoid the awkwardness of face-to-face communication. In addition, the platform can be used to connect with other young people who have had similar experiences. A peer who has suffered a concussion and subsequently recovered will be a valuable resource. However, it is extremely important to maintain the security of the platform network and avoid interference from inappropriate information.

#### 3.3.2 Integrated outcome 2: needs and challenges of adolescents with mBTI during the recovery period

##### 3.3.2.1 Category 6: Ways to obtain academic accommodations

Academic accommodation refers to adolescents having more choices in their academic pursuits, such as fewer learning tasks, more rest time, or separate exam venues. However, such accommodations are often limited and difficult to obtain. Some teachers do not trust students’ self-reports (“I remember arguing with the school because they did not believe I had memory problems unless I could prove it on paper, like taking a memory test”) (15). Some teachers are also unable to provide long-term academic accommodations due to external pressures or their limitations (“I can sense their struggle—it is not that the teachers do not want to help, but they cannot… because we have to adhere to so many standardized rules”) (9). Some adolescents also fail to advocate for themselves, either because they were unaware of the process or distrusted their teachers (“My counselor is disorganized; even if I do not get support from other teachers, it is better than spending a month trying to find him”) (13). Healthcare professionals can provide medical assistance to help adolescents obtain academic accommodations, but implementation varied (“My English teacher failed me in the first semester—he did not believe I was hospitalized, even after the doctor had spoken to the school”) (18). The process of obtaining academic accommodations is fraught with uncertainty, communication between adolescents and teachers is not going smoothly, and there is no comprehensive policy in place to help them.

##### 3.3.2.2 Category 7: Mastering disease information and knowledge

Following mTBI, some adolescents experience symptoms like amnesia, making them eager to understand what happened. (“You have no idea what happened, and it seems like no one can. I do not know if I was different when I arrived”) (18). The treatment process for mBTI is also unsatisfactory (“My parents were not sure what to do, neither did the doctors. I just went to school and had to rely on painkillers to cope with it”) (12). Adolescents reported that healthcare providers’ education on care and exercise recommendations were crucial, but sometimes the advice was ambiguous and hollow (“The doctor told me what I could not do, but did not tell me what I could do” (13)) (“The rehabilitation handbook lacks humanity. I would rather be treated as a person than a number” (12)). Some adolescents mentioned that because people around them knew little about mTBI, it was difficult for them to get timely and effective help (“When I got hit, I did not realize a concussion, so I proceeded with the game, which probably made things worse”) (12). They suggested that schools should use videos, presentations, and other forms of centralized education (e.g., “A video or cartoon… can show how someone with a concussion struggles in school and how friends or peers can help, or how to recognize if someone has a concussion”) (15). The characteristics of mTBI make the process of collecting medical history uncertain. In many cases, there is no opportunity to conduct a systematic medical assessment, and treatment lacks specificity. For adolescents, there is no established education system for such accidents, and self-rescue and rescue efforts after an accident cannot be carried out smoothly.

##### 3.3.2.3 Category 8: Selfless help from friends and family

Family and friends are important sources of social support for adolescents with mTBI, including emotional support, instrumental support, and informational support. Adolescents mentioned that classmates and friends could understand their fatigue and stress help them complete teamwork (“I did less work than usual, and they helped me solve problems and correct my assignments”) (15), ensure they did not forget class materials, and secure their safety in school (“They watched out for me to see if I tripped or felt dizzy”) (11). They also joined teachers in discussing academic accommodations and helped adolescents secure their rights. When adolescents were misunderstood, they stood up for them, offering explanations and arguments. They considered the limitations of adolescents’ activities when making plans and provided ample emotional support (“I loved their surprise visits—I did not know they had come after playing basketball… I was so happy”) (15). While peer support is important, parental assistance is more practical, such as researching healthcare options and health resources, arranging medical appointments, and strategically communicating with school administrators to advocate for academic accommodations (“Parents can communicate with schools better than my friends. My friends are not my guardians and they do not have adult authority”) (13). In addition, parents prioritized their children’s health, allowing them to stay home and helping with homework (“My mom helped me a lot—since I struggled with screens, she emailed my teachers; I dictated, while she typed”) (11). Adolescents described that the sense of security provided by parents was irreplaceable (“I was terrified and in so much pain—I needed my mom by my side all week. She slept in the rocking chair next to me”) (18). Indeed, family and friends play a key role in the support system for adolescents. However, from another perspective, community and social network support are rarely mentioned by adolescents. This warrants careful consideration, exploration of the underlying reasons, and the implementation of appropriate measures.

##### 3.3.2.4 Category 9: Timely and effective treatment and high-quality rehabilitation services

The treatment options for mTBI are relatively limited, primarily consisting of symptomatic medications such as pain relievers and anti-vertigo drugs, with varying degrees of effectiveness depending on the individual. Many adolescents reported long waiting times in emergency rooms after injuries, and doctors provided overly generalized information and very limited treatment options (“I just wanted to cure my headache, but nothing they gave me worked. I stayed in the hospital for a few nights, yet there was still no relief”) (18). Rehabilitation strategies for mTBI mainly include cognitive and motor recovery training. This often requires multidisciplinary teamwork of neurologists, rehabilitation specialists, and psychologists, but this seems difficult to achieve. In the survey, adolescents did not establish long-term effective relationships with professionals (“After admission… I even did not know the doctor’s name—completely anonymous”) (18). They noted that physical and occupational therapies were often provided too late and lacked specificity, expressing a need for more detailed guidance (“I appreciated one nurse who gave practical advice, including concrete examples of optimal self-management within my capabilities”) (13). Adolescents preferred professionals who showed genuine interest in their needs rather than just focusing on the injury (“While conducting tests, she talked with me—unlike others who just did their job and left. We discussed a lot, and it was cool”) (18).

#### 3.3.3 Integrated outcome 3: self-management experience during the mTBI recovery period

##### 3.3.3.1 Category 10: Control of the environment

Adolescents indicated that their requirements on the environment rested with reducing noise and light (“I would go to a quiet, slightly dark place”) (16), far away from crying infants (“That lady kept coming back with puzzles and baby stuff…it got annoying over time”) (18). From a psychological perspective, adolescents’ sense of psychological security and self-efficacy is closely tied to the predictability and manageability of their surroundings. Unlike younger children, they did not desire constant attention; instead, they wanted to escape adult supervision and restrictions… A relatively independent space and sense of control were beneficial. They could decide how to spend their time and access age-appropriate entertainment (“I enjoyed coffee and video games they bought, which helped pass the time”) (18). Although it is beneficial for adolescents to control their environment, this places higher demands on guardians. How to communicate effectively, avoid excessive interference, understand adolescent psychology, and respect their personalities and views. These issues require more theory and practice.

##### 3.3.3.2 Category 11: Adjustment of rest and activity strategies

During mTBI recovery, the most common advice adolescents received was to take a complete break from physical and cognitive activities, followed by a gradual resumption of activities. However, since the advice given by doctors was not clear, each person’s rest and activity strategy differed. Some believed it was necessary (“I will not go back to school until I feel completely better”) (12), They argued that boredom might lead to engaging in activities detrimental to mTBI recovery (“Playing video games until I feel dizzy and then falling asleep”) (17). Adolescents believed they needed to engage in activities to help them manage their energy, like using schedules to plan daily tasks (“Listening to audiobooks and playing with pets”) (16). Moreover, they reintegrated meaningful physical activities into their lives, as exercise not only restored energy but also stabilized mood and improved sleep quality. Overall, relative rest can reduce overall brain metabolism and is more conducive to recovery than complete rest. The type and intensity of activities need to be further standardized. It is worth emphasizing that when adolescent athletes are forced to withdraw from sports due to rest regulations, they should maintain contact with their teammates and coaches. They need additional social support and intervention.

##### 3.3.3.3 Category 12: Positive self-healing

Self-healing involves observing the relationship between the mind and body. By accepting oneself, thinking positively, and regulating emotions, one can restore mental and physical health. Adolescents with mTBI described that they relieved stress through physical relaxation (massage, acupuncture, bathing (16)). They also often engaged in self-counseling through reflection and positive psychological suggestions (“overcome it,” “endure the pain,” “calm yourself down,” “pray” (16)). Some viewed mTBI as a life medal (“They tend to think of me as a child who has had more concussions than most. Compared to other things I have done, this is what I am more known for”) (12), while others channeled anxiety into motivation, came out of from anxiety and depression after deep reflection, and achieved a change in mindset (“During the break, I truly reflected on who I am… It made me a better person, pulling me out of the darkness”) (12). Self-healing among adolescents largely involves sharing simple experiences that are easy to implement but highly random. However, providing professional psychological guidance tailored to the characteristics of adolescents, such as rational emotive therapy and relaxation therapy, can be more effective in helping them restore their physical and mental well-being. Compared to neurological symptoms, mental health has not received sufficient attention, professional psychological services are not widely available, and the mental health education system needs to be improved.

## 4 Discussion

### 4.1 Continuously updated techniques and concepts are the prerequisites for the rehabilitation of adolescents with mTBI

The various difficulties faced by adolescents with mTBI are related to physical symptoms that cannot be alleviated. Timely and accurate medical diagnosis and treatment are crucial, as they are closely associated with faster recovery from mTBI (19). Healthcare professionals should continuously update their diagnostic and treatment techniques and concepts, promptly implement early clinical management, and help adolescents and their caregivers understand the condition rationally and correctly.

From traditional experience, though head computed tomography (CT) scans are an important diagnostic tool for mTBI, the decision-making process for CT scans is inefficient and carries radiation risks. The Canadian CT Head Rule (20) can reduce unnecessary CT examinations and help doctors determine whether imaging is necessary for adolescents through the Glasgow Coma Scale (GCS), signs of skull base fractures, and frequency of vomiting, thereby lowering radiation risks and medical costs. In actual clinical practice, the symptoms of mTBI are often subtle and transient, and influenced by numerous confounding factors, such as traumatic stress, other injuries, and medication use, which can affect medical evaluation and delay diagnosis. Therefore, the American Congress of Rehabilitation Medicine Diagnostic Criteria for mTBI states (3) that when a patient meets the suspected mTBI criteria, treatment should follow the principle of rest immediately if suspected, with a stepwise plan for resuming activities as required. The International Traumatic Brain Injury Research initiative has demonstrated that blood biomarkers during the acute phase post-injury can help reduce unnecessary CT scans and hospitalizations (21). Visser K (22) demonstrated that as a result of inflammatory responses after mTBI, interleukin-6 was the most promising biomarker for clinical diagnosis of brain injury, while interleukin-10 was a potential candidate for CT scan triage. Other studies have confirmed that S100B protein is a diagnostic tool and a possible therapeutic target for mTBI (23, 24). In terms of imaging, Chinese researchers designed a framework mTBI-DSANet, which uses resting-state functional magnetic resonance imaging (rs-fMRI) to collect data and appraise performance, demonstrating its potential in diagnosing mTBI (25). Based on the above principles, doctors should follow four core principles in diagnosis and treatment, namely, accurate physical examinations and imaging decision-making, rapid screening using biomarkers, stepwise rehabilitation management, and control of confounding factors. Key factors for the nursing team include observation of changes in intracranial pressure, quantitative recording of symptoms, control of the environment, and management of body position.

### 4.2 Assessment, screening, and intervention of adolescent mental health are critical to mTBI rehabilitation

Integrated outcomes found that anxiety in adolescents with mTBI can exacerbate symptoms and even cause post-concussion syndrome, consistent with IversonGL’s research results (26). Therefore, importance should be attached to the assessment and screening of adolescent mental health. Common scales for mental health comprise the depression module of the Patient Health Questionnaire (PHQ-9) and the post-traumatic stress disorder checklist. Additionally, the prospective study by Gornall A (27) also proved that pre-injury demographic and psychological factors in adolescents could predict post-concussion mental health problems. This also reminds healthcare personnel to value the past medical history of adolescents. When anxiety occurs, mind–body interventions can be a more effective alternative to psychiatric drugs (28). For example, relaxing the body through sensory contact with the natural environment can alleviate anxiety and post-concussion syndrome (29). Healthcare workers should maintain a positive attitude during interactions. The fear-avoidance model is a valuable perspective that can help us learn about the life experiences of adolescents who have recently suffered a concussion and also experienced anxiety and focus on the interactions of concussion manifestations, anxiety, and pain-related fears, providing adaptive coping strategies (30). Meanwhile, the importance of continuous assessment cannot be ignored. The Depression, Anxiety and Stress Scale-42 (DASS-42) and the World Health Organization Quality of Life-BREF (WHQOL-BREF) can describe the recovery trajectory of this population and guide intervention strategies (31). In addition to medical intervention, the family should be a core component of adolescent mental health intervention. Family members can help adolescents adjust their physical environment and manage their time and participate in family cognitive behavioral therapy, acting as cognitive collaborators, behavioral coaches, and emotional containers. Therefore, family intervention should be incorporated into the standardized process of clinical pathways, and a collaborative mechanism should be established with hospitals.

### 4.3 Promoting academic accommodations for adolescents with mTBI to help them return to school

Integration results found that adolescents experienced academic difficulties after mTBI and took a longer time than children to return to school (32). This may be linked to their higher academic requirements, greater sensitivity to disease-related academic losses and time costs, and heavier psychological burden, consistent with PeiY’s (33) systematic evaluation results. Academic accommodations are crucial for adolescents to return to school (34), but the process is often fraught with obstacles. To this end, a long-term educator resource library SCHOOL First has been developed, which can help school workers understand concussions and how to advocate for academic accommodations (35). McAvoyK (36) also explored a community healthcare system to train school professionals through remote guidance. This model enabled school professionals to offer knowledge and self-efficacy and increase communication and cooperation among families, schools, and healthcare providers. Specifically, in this model, medical institutions share dynamic rehabilitation data through the system, such as daily cognitive measurement data, schools can use decision tree tools to convert clinical opinions into teaching plans, and community workers can monitor homework completion and changes in adolescents’ emotions through the platform.

Besides, schools should incorporate concussion knowledge into teaching plans to cultivate students’ awareness of self-protection and disease-coping strategies. For example, adolescents are taught how to correctly describe their pain and exhaustion, and to explain invisible injuries to classmates and friends, like using mobile phone battery loss as a metaphor for their energy level after mTBI. Schools can also carry out simulation drills to enable students to master the correct emergency response methods through practice, thus effectively helping injured classmates. Finally, the application process for academic accommodations should be streamlined. Healthcare institutions can download a unified letter from the CDC Heads Up website to describe students’ symptoms, expected accommodations, or reasons for absence. Future tools should be effectively integrated into the medical system, interact with adolescents in a user-friendly manner, make medical certificates more accessible, facilitate communication between all parties, and reduce the difficulty of self-advocacy for academic accommodations (37).

### 4.4 Respecting the individual needs of adolescents with mTBI and providing scientific management guidance through community medical services

The integrated results found that adolescents wanted to be involved in decisions about their treatment and care, consistent with relevant research results of adolescents with other diseases (38). Notably, the strategies offered by adolescents even exceeded the recommendations of expert consensus, exhibiting strong subjectivity and instability. Therefore, the rehabilitation process requires regular supervision and follow-up by the community medical team (39). The primary care units in the community should be equipped with nurses specializing in concussions. They should implement a closed-loop service consisting of “decision assessment, joint consultation on treatment plans, and supervision of implementation.” Electronic health records for mTBI should be established to enable cross-institutional data sharing and link with a database of neurological specialists. Specifically, community health workers should help adolescents screen and collect scientific mTBI online educational resources among the vast amount of information available, based on the latest evidence, best practices, and consensus statements, like the Brain Injury Association of America (40) and Centers for Disease Control and Prevention (CDC) Concussion and mTBI website (41). Furthermore, they should assist adolescents in choosing applicable management tools. For example, a Self-Monitoring Activity-Restriction and Relaxation Training (SMART) program has an interactive design and gamified components, which make it easier for adolescents to accept and master (42), thus providing potential, extensible, and anonymous treatment and support. Medical social workers can help families in need conduct simple furnishings, for instance, utilizing assistive tools like non-slip mats, movable shower handrails, and shower chairs; relocating items with simple signs to help adolescents find and take them; adjusting the color of home lights, applying soundproof seals to block noise, and preventing sound and light stimulation. At present, China is rapidly promoting family doctor contract services (43). A comprehensive service team should be established with family doctors as the principal part, together with neurosurgery, neurology, psychology, and rehabilitation departments of higher-level medical institutions, to help mTBI adolescents from different perspectives.

### 4.5 Limitations

This paper systematically reviewed eight qualitative studies on the rehabilitation experiences and needs of adolescents with mTBI. After manual searching, a meta-synthesis was conducted on various databases. The synthesis process was rigorous, with a total of eight high-quality articles included. Six of these articles were rated as Grade B in quality. Three of the six articles did not indicate whether they complied with current ethical standards. The other three articles did not explain the researchers’ circumstances, the researchers’ influence on the research, or the research’s influence on the researchers from the perspective of cultural background and values. However, considering the professionalism of the authors’ team and the high-quality research results, this impact can be ignored, and the results can be used as a basis for evidence-based practice. Nevertheless, it cannot be denied that this study has several limitations. First, according to the inclusion criteria for the literature, only qualitative studies published in indexed journals and written in English were selected. Therefore, gray literature and some papers or theses were not retrieved, which may lead to information bias. In addition, due to cultural and policy differences, the lifestyles, cultural interests, and psychological qualities of adolescents in different countries and regions are not entirely the same. Different researchers have different understandings and interests. Furthermore, adolescents’ academic pressure, social resources, and values are greatly influenced by the cultures of different regions. Therefore, the results of this study may have certain limitations.

## 5 Conclusion

This study adopted a meta-synthesis approach to discuss the rehabilitation experience of adolescents with mTBI and comprehensively investigate their symptoms, dilemmas, needs, challenges, and self-management experience during the rehabilitation period. Healthcare professionals should realize that mTBI symptoms are complex and interactive, and adolescents need more timely and accurate medical services, more systematic and comprehensive social support, and more independent disease management strategies. Therefore, interdisciplinary team collaboration can minimize the risk of injury in adolescents, which relies on the construction of a close-knit medical alliance and the continuous improvement of the community medical service system. Apart from that, further exploration and practices are required to determine how the healthcare and education systems can collaborate more effectively to help adolescents return to school and resume their studies.
